# The relationship of physical activity and sedentary behavior patterns with osteoporosis in older women: a dose–response analysis

**DOI:** 10.3389/fpubh.2026.1717573

**Published:** 2026-01-29

**Authors:** Xin Li, Bowen Li

**Affiliations:** College of Sports Science, Tianjin Normal University, Tianjin, China

**Keywords:** dose–response analysis, older women, isotemporal substitution, osteoporosis, physical activity, sedentary behavior

## Abstract

**Objective:**

Osteoporosis frequently affects older women and is strongly linked to their daily routines, which include both sedentary behavior (SEB) and physical activities (PA) of different intensities. This study investigates the dose–response relationship of different SEB and PA patterns among community-dwelling older women and assesses the potential impact of time reallocation on osteoporosis risk through an isotemporal substitution analysis.

**Methods:**

In this study, 1,106 older women aged between 60 and 70 years in Tianjin participated. Their moderate to vigorous physical activity (MVPA), light physical activity (LPA), and SEB were objectively assessed using an accelerometer. The connection between MVPA, LPA, SEB, and osteoporosis was assessed using binary logistic regression models and isotemporal substitution models.

**Results:**

The osteoporosis group and non-osteoporosis group comprised 461 and 645 subjects respectively, accounting for 41.68 and 58.32% of the total cohort. The osteoporosis group had significantly higher daily SEB compared to the non-osteoporosis group (*p* < 0.01), whereas LPA (*p* < 0.05) and MVPA (p < 0.01) were notably lower. Dose–response analysis adjusted for variables revealed: increases of 60 min/d in total SEB, as well as 10-min, 30-min, and 60-min SEB bouts, were positively correlated with the risk of osteoporosis. While increases of 30 min/d in total, sporadic, and bouted LPA were negatively associated with osteoporosis risk. More notably, adding 10 min/d to total, sporadic, and bouted MVPA significantly decreased the risk of osteoporosis (*p* < 0.01). Isotemporal substitution model analysis demonstrated: replacing 30 min of SEB with equivalent durations of LPA and MVPA increased osteoporosis risk by 9 and 56%, respectively. Substituting 30 min of SEB with equivalent durations of LPA and MVPA reduced osteoporosis risk by 8 and 47%, respectively. Switching 30 min of LPA with the same duration of MVPA reduced osteoporosis risk by 28%, while exchanging 30 min of MVPA with the same duration of LPA showed no significant difference.

**Conclusion:**

PA and SEB in older women exhibit a significant dose–response relationship with osteoporosis. Avoiding prolonged sitting and increasing PA duration both offer protective effects against osteoporosis in older women, with achieving a certain level of MVPA being the most effective protective measure.

## 1 Introduction

China is facing a severe aging society. Aging will have profound impacts on China’s macroeconomic operations, social welfare systems, and other areas. Improving the health of the older adults is crucial for the stability and sustainable development of society as a whole. As physiological age advances, numerous age-related diseases emerge—such as osteoporosis, hypertension, and coronary heart disease—increasing pressure on healthcare systems and hindering healthy aging. Thus, achieving healthy aging has become an imperative societal challenge. The incidence of osteoporosis in the older adults has consistently increased over the past twenty years, now affecting about one-third of them (1).

Bone strength is largely determined by bone mineral density and the quality of the bone. Factors influencing bone quality mainly include bone remodeling capacity, bone microarchitecture, and bone mineralization. Internationally, osteoporosis is etiologically classified into three major categories: primary, secondary, and idiopathic osteoporosis. Primary osteoporosis represents an inevitable physiological degenerative process associated with aging, subdivided into postmenopausal osteoporosis and senile osteoporosis. Postmenopausal osteoporosis occurs when estrogen levels rapidly decline after menopause, leading to accelerated bone resorption exceeding formation and accelerated bone mass loss. Senile osteoporosis describes age-related conditions where bone mass per unit volume falls below normal levels, accompanied by increased trabecular porosity, reduced bone matrix, and diminished bone strength. Osteoporosis compromises bone structure in the older adults, elevates fracture risk, diminishes muscle strength, causes pain and numbness in various body parts, and lowers quality of life (2). Furthermore, in the older adults, osteoporotic fractures are strongly connected to increased mortality rates and greatly hinder physical mobility.

Osteoporosis is a major health issue for older women, characterized by high prevalence and incidence. In a study involving 207 post-menopausal Moroccan women, 18.3% were found to have densitometric osteoporosis, while vertebral fractures were identified in 62.3% of the participants (3). The results demonstrate a significant occurrence of osteoporosis and associated fractures within this population. Similarly, in a retrospective study of the Swiss Evaluation of the Methods of Measurement of Osteoporotic Fracture Risk, among 556 post-menopausal women, the incidence of fragility fractures was 9.4% after an average follow-up of 2.7 ± 0.8 years (4). The Hertfordshire Cohort Study, which examined 498 men and 498 women aged 59 to 72, found that risk factors for poor bone health, like low physical activity, frequently appeared together. Among women, a graded correlation was identified between the number of risk factors and reduced bone density, and there were also strong ties between the number of risk factors and fracture incidents. An adjusted hazard ratio of 5.98 for incident fractures was observed in women with three or more risk factors, as opposed to those with none (5). This study showed that physical activity is vital for bone health and that its absence, combined with other risk factors, could lead to a higher risk of fractures in older women.

PA is considered a key factor in maintaining bone health (6). By adjusting individual lifestyles before disease onset, PA maximizes health benefits and represents the optimal approach to achieving exercise-induced health gains (7). Simultaneously, the health benefits of PA for enhancing bone health have been confirmed by numerous studies. Multiple evidence sources recommend exercise programs for postmenopausal osteoporosis patients that prioritize resistance training supplemented by aerobic and balance exercises (6–8). Muir’s osteoporosis research found that increased PA positively impacts bone density in the hip, Ward’s triangle, femoral neck, and trochanter (9). As research deepens, studies targeting older adults further emphasize the importance of PA for bone health.

SEB has been demonstrated as a risk factor for the onset and progression of multiple diseases (10–13), with studies showing an association between SEB and lower bone mineral density (14). The amount of time women spend being sedentary is negatively associated with their total femoral and segmental bone mineral density, independent of their involvement in MVPA and vigorous physical activity (VPA) (15). The connection between PA and SEB with health outcomes can be studied through a pattern-based approach. However, current studies on PA and SEB patterns are sparse, and only a few have utilized objective methods to examine the dose–response relationship between these patterns and osteoporosis in older females.

Against the backdrop of developing physical activity guidelines for older women and intensifying research on PA and SEB, investigating the intrinsic relationship between PA, SEB and osteoporosis in older women is crucial for helping them achieve their fitness goals. This study aims to facilitate the practical translation and dissemination of research findings, enhance the physical health of older women, promote healthy aging, and contribute to a healthier society.

## 2 Methods

### 2.1 Participants

This study recruited 1,198 older individuals from communities in Tianjin. Participants were women aged 60–70 years who could communicate independently. Exclusion criteria for participant screening included: individuals suffering from diseases that could affect the metabolism of bone or calcium; those with foot injuries preventing heel bone density testing; those taking medications that could affect bone or calcium metabolism, such as calcitonin or bisphosphonates; individuals with cognitive impairment; those with physical disabilities preventing participation in standard physical activities; and those who failed to complete the study’s testing protocols. A total of 92 participants were excluded, resulting in 1,106 eligible participants. All participants voluntarily signed informed consent forms after receiving full disclosure of the study’s objectives.

### 2.2 Study design

This study aims to examine the relationship between PA, SEB, and physical health in older women. Participants completed questionnaire surveys and experimental indicator tests. The questionnaire survey method was employed to collect participants’ demographic information, personal lifestyle habits, and physical and mental health-related data. An experimental approach was employed to obtain anthropometric measurements (height, weight), grip strength test results, bone density data, PA duration and intensity, and SEB duration. Data underwent double-entry and double-verification procedures before being organized and imported into Stata 16.0 software for statistical analysis, ultimately yielding the study conclusions.

### 2.3 Variables and measuring instruments

#### 2.3.1 Basic information of participants

The questionnaire collected participants’ sociodemographic characteristics (age, education level, cohabitation status), personal lifestyle habits (alcohol consumption, tea drinking), and information related to health (quality of life, nutritional status, cognitive function, and insomnia). Health-related quality of life was assessed using the Chinese version of the European Quality of Life 5-Dimensional Instrument (EQ-5D) (16). Nutritional status was evaluated with the Mini Nutritional Assessment (MNA) (17), and cognitive function was assessed using the Mini-Mental State Examination (MMSE) (18) and the Athens Insomnia Scale (AIS) to assess insomnia status (19). Muscle mass and body mass index (BMI) were measured using a body composition analyzer (Tanita MC-180, Japan) via multi-frequency bioelectrical impedance analysis. Grip strength was measured using an electronic dynamometer (WCS-100, China).

#### 2.3.2 PA and SEB

PA and SEB were measured using a triaxial accelerometer (Actigraph wGT3X-BT, Pensacola, FL, USA). Participants were directed to have the accelerometer on their left hip for 7 consecutive days, not including when they were asleep, bathing, or swimming, and maintaining their usual lifestyle habits. The 7-day wear period included both weekdays and weekends (20). Experimental staff conducted regular telephone follow-ups to remind participants to wear the accelerometer as instructed, ensuring data completeness and validity. Raw accelerometer data was sampled at 30 times per second. This raw data was imported into Actilife software and converted into a 60-s sampling interval format. Valid data were defined as daily wear time ≥10 h and ≥4 days of wear, with no requirement for weekdays versus weekends within the valid wear period (21, 22). Non-wear time is characterized by at least 90 continuous minutes where the accelerometer records zero counts, allowing for up to 2 min with counts under 100 counts per minute (23, 24). SEB, LPA, and MVPA were classified using Freedson’s threshold values: SEB (0–99 counts/min); LPA (100–1,951 counts/min); MVPA (≥1,952 counts/min) (25). The sum of SEB, LPA, and MVPA was used to calculate the total accelerometer wear time. To further analyze the relationship between PA and SEB patterns and osteoporosis, SEB, LPA, and MVPA were subdivided into more specific categories. Specific criteria are detailed in Table 1.

**Table 1:** Criteria for sedentary behavior and physical activity patterns.

Behavioral patterns	Standard
10 min-Bouted SEB	Single session duration SEB ≥ 10 min
30 min-Bouted SEB	Single session duration SEB ≥ 30 min
60 min-Bouted SEB	Single session duration SEB ≥ 60 min
Sporadic LPA	Single session duration LPA < 10 min
Bouted LPA	Single session duration LPA ≥ 10 min
Sporadic MVPA	Single session duration MVPA < 10 min
Bouted MVPA	Single session duration MVPA ≥ 10 min

#### 2.3.3 Bone mineral density measurement

Bone mineral density was measured using an ultrasonic bone densitometer (Sonost-2000, Korea). First, connect the device to the computer, launch the Sonost 2000 software, and perform calibration before use. After connecting the device to the compatible computer and opening the Sonost-2000 program, use the test module for daily calibration. Once calibration is complete, the device is ready for use. Prior to testing, inquire about the subject’s age, gender, and other relevant information, then input these details into the computer. Instruct the subject to remove the shoe and sock from their left foot. Apply medical ultrasound coupling gel evenly to the skin on both sides of the left heel bone. Position the left foot in the instrument’s testing slot, ensuring the heel makes full contact with the lower part of the device. Once the heel bone is directly aligned with the two side probes, initiate the measurement of the bone mineral density T-score.

### 2.4 Statistical analyses

Data were imported into Stata 16.0 for descriptive statistical analysis. Continuous variables were tested for normality. Those meeting normality assumptions underwent independent samples *t*-tests, while Wilcoxon rank-sum tests were used to analyze continuous variables that were not normally distributed. Chi-square tests were used to evaluate categorical variables and identify differences between the osteoporosis and non-osteoporosis groups. The mean and standard deviation represent continuous variables, while categorical variables are expressed as frequency and percentage (percentage).

A binary logistic regression analysis with variable adjustments was performed to explore the dose–response relationship between various PA and SEB patterns, their isotemporal substitution model, and osteoporosis in older women. This study set incremental independent variables: total SEB, 10 min-Bouted SEB, 30 min-Bouted SEB, and 60 min-Bouted SEB increased by 60 min/d; total LPA, Sporadic LPA, and Bouted LPA increased by 30 min/day; total MVPA, Sporadic MVPA, and Bouted MVPA increased by 10 min/day (26, 27). Investigate the dose–response dynamics among various PA (total LPA, Sporadic LPA, Bouted LPA, total MVPA, Sporadic MVPA, Bouted MVPA), SEB (total SEB, 10-min Bouted SEB, 30-min Bouted SEB, 60-min Bouted SEB) and osteoporosis in older women. Using the isotemporal substitution model, the absolute time reallocation of SEB, LPA, and MVPA was analyzed by substituting time from one activity to another, to determine its association with osteoporosis status. Two types of binary logistic regression models, a univariate model and an isotemporal substitution model, were applied to analyze the connection between SEB, LPA, and MVPA with osteoporosis. The univariate model assessed the relationship between increasing any single activity component, rather than substitution, and osteoporosis. The isotemporal substitution model evaluated the relationship between substituting one activity type for another with equal time expenditure and osteoporosis. The outcomes of the logistic regression were presented as odds ratios (OR) along with 95% confidence intervals (95% CI). The variance inflation factor (VIF) was calculated for all variables to detect multicollinearity, with a VIF < 5 for each covariate (28). For two-tailed tests, statistical significance was determined at a *p*-value of less than 0.05.

### 2.5 Quality control

To ensure survey quality, this study provided standardized training for all participants. After each survey session, data was uniformly entered and lessons learned were summarized to promptly address any issues encountered. Questionnaires were administered through one-on-one interviews, with interviewers using standardized explanations to ensure participants fully understood each question before making selections. Questionnaires were distributed and collected on-site. During accelerometer wear, investigators provided standardized instructions on usage, requirements, and precautions. Daily one-on-one telephone follow-ups were conducted, primarily addressing accelerometer wear status and participant inquiries. Body composition analyzers, electronic grip strength meters, and ultrasonic bone density testers were inspected and disinfected before each use. Measurements strictly adhered to operational protocols to ensure accurate and safe experimental procedures. After testing concluded, all measurement instruments were recalibrated and disinfected. Data entry and verification were performed by two individuals to ensure accuracy.

## 3 Results

### 3.1 Descriptive analysis

The study initially recruited 1,198 older women, excluding 92 who did not meet the analysis criteria, resulting in a final sample of 1,106 subjects for analysis. There were 461 individuals in the osteoporosis category and 645 in the non-osteoporosis category, which correspond to 41.68 and 58.32% of the participants, respectively. Between the osteoporosis and non-osteoporosis groups, significant differences were noted in BMI, muscle mass, grip strength, and bone mineral density T-scores (*p* < 0.05). Age, education, living arrangements, alcohol and tea intake, EQ-5D, MNA, MMSE, and AIS assessments showed no significant differences between the groups (Table 2).

**Table 2:** Characteristics of participants.

Variable	Total (*N* = 1,106)	Osteoporosis		*p*
		Yes (*N* = 461)	No (*N* = 645)	
Age (year)	64.91 ± 3.57	65.18 ± 3.82	64.72 ± 3.36	0.679
Living alone, *N* (%)				0.452
Yes	127 (11.48)	49 (10.63)	78 (12.09)	
No	979 (88.52)	412 (89.37)	567 (87.91)	
Educational level, *N* (%)				0.521
Primary school and below	129 (11.66)	51 (11.06)	78 (12.09)	
Middle school	781 (70.61)	334 (72.45)	447 (69.30)	
University and above	196 (17.72)	76 (16.49)	120 (18.60)	
Tea drinking, *N* (%)				0.814
Yes	324 (29.29)	133 (29.07)	191 (29.61)	
No	782 (70.71)	327 (70.93)	455 (70.93)	
Alcohol drinking, *N* (%)				0.908
Yes	109 (9.86)	46 (9.98)	63 (9.77)	
No	997 (90.14)	415 (90.02)	582 (90.23)	
BMI (kg/m^2^)	25.37 ± 3.42	25.72 ± 3.81	25.12 ± 3.27	0.039
Muscle mass (kg)	38.62 ± 3.59	38.14 ± 3.32	38.96 ± 3.76	0.046
Grip strength (kg)	24.13 ± 2.84	23.77 ± 2.65	24.39 ± 2.97	0.034
EQ-5D Assessment	78.27 ± 8.53	77.91 ± 8.02	78.53 ± 8.85	0.328
MNA Assessment	13.39 ± 1.26	13.29 ± 1.09	13.46 ± 1.48	0.087
MMSE Assessment	26.73 ± 2.41	26.65 ± 2.38	26.79 ± 2.52	0.394
AIS Assessment	2.07 ± 0.36	2.20 ± 0.43	1.98 ± 0.33	0.073
Bone mineral density T value	−2.19 ± 0.25	−2.81 ± 0.28	−1.75 ± 0.23	<0.001

### 3.2 Relationship between different behavioral patterns and osteoporosis in older women

Table 3 indicates that the subjects in this research had an average accelerometer wearing time of 886.57 min/d, among which the average duration of Total SEB was 546.28 min/d, accounting for 61.62% of the daily accelerometer wearing time. A significant difference was found between the osteoporosis group, with 63.88% Total SEB, and the non-osteoporosis group, which had 60.00% (*p* < 0.01). In comparison to the non-osteoporosis group, the osteoporosis group had SEB durations of 354.17 min/d for 10 min-Bouted SEB, 182.59 min/d for 30 min-Bouted SEB, and 83.95 min/d for 60 min-Bouted SEB, representing increases of 5.51, 13.09, and 25.69%, respectively (*p* < 0.01).

**Table 3:** The relationship between different behavioral patterns and osteoporosis in older women.

Variable (min/d)	Total (*N* = 1,106)	Osteoporosis		*p*
		Yes (*N* = 461)	No (*N* = 645)	
Accelerometer wear time	886.57 ± 90.31	885.93 ± 92.59	887.03 ± 93.62	0.819
Total SEB	546.28 ± 60.84	565.89 ± 62.07	532.26 ± 59.18	0.004
10 min-bouted SEB	343.39 ± 38.16	354.17 ± 39.82	335.69 ± 36.57	0.005
30 min-bouted SEB	170.26 ± 20.69	182.59 ± 22.46	161.45 ± 18.06	0.002
60 min-bouted SEB	73.94 ± 8.37	83.95 ± 10.07	66.79 ± 7.64	<0.001
Total LPA	305.76 ± 42.16	293.64 ± 39.61	314.42 ± 45.32	0.027
Sporadic LPA	123.19 ± 13.52	117.93 ± 12.73	126.95 ± 13.94	0.018
Bouted LPA	182.57 ± 19.76	175.71 ± 18.69	187.47 ± 20.43	0.031
Total MVPA	34.53 ± 4.93	26.40 ± 3.87	40.35 ± 5.69	<0.001
Sporadic MVPA	23.95 ± 3.07	19.13 ± 2.83	27.39 ± 3.64	<0.001
Bouted MVPA	10.58 ± 1.32	8.27 ± 1.16	12.96 ± 1.57	<0.001

Regarding LPA, the mean Total LPA duration for all subjects was 305.76 min/day, accounting for 34.49% of the daily accelerometer wear time. In the osteoporosis group, the average Total LPA duration was 293.64 min/day, with Sporadic LPA and Bouted LPA constituting 40.16 and 59.84% of Total LPA duration, respectively. The osteoporosis group exhibited significantly lower durations of Total LPA, Sporadic LPA, and Bouted LPA compared to the non-osteoporosis group, with reductions of 6.61, 7.11, and 6.27%, respectively (*p* < 0.05).

In terms of MVPA, the average duration of Total MVPA of the subjects was 34.53 min/day, accounting for 3.89% of the daily accelerometer wear time. The non-osteoporosis group had a mean total MVPA duration of 40.35 min/d, significantly higher than the 26.40 min/d in the osteoporosis group (*p* < 0.01). For the durations of Sporadic MVPA and Bouted MVPA, those in the non-osteoporosis group were 27.39 min/d and 12.96 min/d respectively, both significantly higher than those in the osteoporosis group by 30.16 and 36.19% (*p* < 0.01).

### 3.3 Dose–response relationship between different behavioral patterns and osteoporosis in older women

Table 4 presents the results of binary logistic regression analyses examining the independent associations between different patterns of PA and SEB and osteoporosis in older women. For SEB, with an increase of 60 min/d, Total SEB, 10 min-Bouted SEB, 30 min-Bouted SEB, and 60 min-Bouted SEB were all significantly correlated with osteoporosis in older women (*p* < 0.05), and the OR values increased by 34, 35, 32, and 31%, respectively. Increasing Total LPA, Sporadic LPA, and Bouted LPA by 30 min/d were significantly associated with osteoporosis in older women (*p* < 0.05), with OR values decreasing by 17% (OR = 0.83, 95% CI: [0.78–0.93]), 13% (OR = 0.87, 95% CI: [0.80–0.97]), and 19% (OR = 0.81, 95% CI: [0.76–0.90]), respectively. All variables of MVPA with an increase of 10 min/d were significantly correlated with osteoporosis in older women. A 10-min daily increase in Total MVPA, Sporadic MVPA, and Bouted MVPA was associated with a 29% reduction (OR = 0.71, 95% CI: [0.62–0.85], *p* = 0.002), 38% (OR = 0.62, 95% CI: [0.49–0.78], *p* = 0.001), and 27% (OR = 0.73, 95% CI: [0.63–0.87], *p* = 0.009), respectively.

**Table 4:** The dose–response relationship between different behavioral patterns and osteoporosis in older women.

Different behavioral patterns	OR (95%CI)	*p*
Total SEB + 60 min/d	1.34 (1.10–1.65)	0.017
10 min-bouted SEB + 60 min/d	1.35 (1.09–1.68)	0.013
30 min-bouted SEB + 60 min/d	1.32 (1.11–1.59)	0.021
60 min-bouted SEB + 60 min/d	1.31 (1.07–1.62)	0.036
Total LPA + 30 min/d	0.83 (0.78–0.93)	0.041
Sporadic LPA + 30 min/d	0.87 (0.80–0.97)	0.048
Bouted LPA + 30 min/d	0.81 (0.76–0.90)	0.037
Total MVPA +10 min/d	0.71 (0.62–0.85)	0.002
Sporadic MVPA +10 min/d	0.62 (0.49–0.78)	0.001
Bouted MVPA +10 min/d	0.73 (0.63–0.87)	0.009

### 3.4 Isotemporal substitution effects of different behavioral patterns on osteoporosis in older women

Table 5 presents the isotemporal substitution effects of PA and SEB on osteoporosis in older women. Both the univariate model and the isotemporal substitution model assessed the impact of PA and SEB on osteoporosis in older women through variable-adjusted binary logistic regression. Results from the univariate model indicate that increasing SEB by 30 min daily significantly increased the OR value for osteoporosis in older women (OR = 1.16, 95% CI: [1.06–1.31], *p* = 0.032). Conversely, increasing LPA and MVPA by 30 min daily reduced the OR value for osteoporosis by 27% (OR = 0.83, 95% CI: [0.78–0.93], *p* = 0.041) and 44% (OR = 0.56, 95% CI: [0.47–0.72], *p* < 0.001), respectively, all with significant differences.

**Table 5:** The effects of 30 min isotemporal substitution of SEB, LPA and MVPA on osteoporosis in older women.

Model	30 min SEB		30 min LPA		30 min MVPA	
	OR (95%CI)	*p*	OR (95%CI)	*p*	OR (95%CI)	*p*
Univariate model	1.16 (1.06–1.31)	0.032	0.83 (0.78–0.93)	0.041	0.56 (0.47–0.72)	<0.001
Replace 30 min SEB	–	–	0.92 (0.88–0.98)	0.042	0.53 (0.45–0.67)	<0.001
Replace 30 min LPA	1.09 (1.02–1.21)	0.013	–	–	0.72 (0.62–0.85)	0.003
Replace 30 min MVPA	1.56 (1.43–1.75)	<0.001	0.96 (0.87–1.12)	0.219	–	–

In the isotemporal substitution model, replacing 30 min of LPA and MVPA with SEB significantly increased the OR values for osteoporosis in older women by 9% (OR = 1.09, 95% CI: [1.02–1.21], *p* = 0.013) and 56% (OR = 1.56, 95% CI: [1.43–1.75], *p* < 0.001), respectively. The OR values for osteoporosis in older women was reduced by 8% (OR = 0.92, 95% CI: [0.88–0.98], *p* = 0.042) and 47% (OR = 0.53, 95% CI: [0.45–0.67], *p* < 0.001) when 30 min of SEB was replaced with equivalent durations of LPA and MVPA, respectively. Substituting 30 min of LPA with the same amount of MVPA led to a 28% reduction in the OR values for osteoporosis in older women (OR = 0.72, 95% CI: [0.62–0.85], *p* = 0.003), while when replacing the same amount of MVPA with 30 min of LPA, there was no significant difference in the OR value of osteoporosis in older women.

## 4 Discussion

This study explored the correlation between behavioral patterns and osteoporosis in older women through dose–response modeling and isotemporal substitution modeling. The primary discovery of the research indicates that diminishing daily SEB and augmenting LPA and MVPA, particularly by reallocating some SEB time to MVPA, represents a more effective approach in mitigating the susceptibility to osteoporosis among older women. The investigation revealed substantial benefits of MVPA in isotemporal substitution assessments, with sporadic MVPA demonstrating a notably pronounced impact in quantitative efficacy evaluations, underscoring the importance of enhancing bone health by integrating MVPA in brief intervals throughout daily routines.

### 4.1 Relationship between behavioral patterns and osteoporosis in older women

Osteoporosis, a highly prevalent disease among the older population, particularly older women, impacts the overall health of the older adults (29). As a preventable and treatable disease, strengthening prevention efforts among high-risk groups can effectively reduce fracture risk (30). Among the 1,106 older female participants in this study, 461 (41.68%) had osteoporosis. Identifying issues in daily behaviors and implementing proactive interventions can effectively improve bone health.

Among older women, maintaining bone health is significantly influenced by PA and SED. A lack of adequate physical activity has been associated with a higher risk of developing and exacerbating chronic diseases like osteoporosis (31). In a Canadian osteoporosis study (10), 1,169 women who are postmenopausal and at least 75 years old provided information about their daily physical activity through a questionnaire. Bone mineral density (BMD) was assessed at five locations using dual-energy X-ray absorptiometry (DEXA): the lumbar spine, femoral neck, total hip, Ward’s triangle, and trochanteric areas. Progressively increasing daily PA was found to positively impact bone mineral density at the hip, Ward’s triangle, femoral neck, and trochanteric sites, according to multivariate linear regression analysis. Xu et al. (32) analyzed 377,234 European participants and found habitual vertical PA increased lumbar spine BMD, while higher overall PA levels correlated with greater forearm BMD. Savikangas et al. (33) examined the connection between PA and osteoporosis in 284 Finnish older individuals aged 70–85 years. PA was assessed using triaxial accelerometers, and femoral bone density was measured via DEXA. Results indicated that LPA is positively correlated with femoral bone density. In a study of German older women, modifiable health risk factors (MHRFs) were analyzed (34). Physical inactivity was one of the MHRFs, and the study identified seven risk clusters. The consistency of these risk clusters among different study groups offers a foundation for specific health interventions. In the context of osteoporosis, interventions aimed at reducing physical inactivity in older women could potentially break these risk clusters and reduce the risk of osteoporosis.

SDB can negatively impact bone health. Compared to standing posture, long-term sedentary behavior reduces musculoskeletal loading, which might cause a reduction in bone density and is not conducive to maintaining bone health (9). Chopra et al. (35) found that the correlation between PA and SDB patterns and BMD in postmenopausal women. The outcomes revealed that in the normal BMD group (BMD T-score ≥ −1.0), 52% of daily sedentary time consisted of SDB episodes lasting over 20 min. In the low BMD group (BMD T-score < −1.0), SDB accounted for 58% of sedentary time. Compared to the normal group, the low BMD group had longer total daily sedentary time (687 min vs. 669 min), fewer sedentary breaks (88 vs. 93), and longer single-session sedentary durations (15.8 min vs. 15.1 min), and less total PA time (249 min vs. 257 min). This aligns with our findings, which revealed that the osteoporosis group exhibited a 6.32% higher daily SEB duration (*p* < 0.01) and LPA and MVPA duration were 6.61% (*p* < 0.05) and 34.57% (p < 0.01) lower, respectively. This indicates a positive correlation between physical activity and bone mineral density, where increased physical activity levels are associated with higher bone density and a reduced risk of osteoporosis. SDB is negatively correlated with bone mineral density, where prolonged SDB duration is associated with reduced bone density and an increased risk of osteoporosis.

### 4.2 Dose–response relationship between different behavioral patterns and osteoporosis in older women

In analyzing the dose–response association between PA patterns and osteoporosis in older females, this study categorized LPA and MVPA into Total LPA, Sporadic LPA, Bouted LPA, Total MVPA, Sporadic MVPA, and Bouted MVPA to further investigate the dose–response correlation between different PA patterns and osteoporosis. The analysis results revealed that all indicators of LPA and MVPA were significantly linked to osteoporosis. Notably, Total MVPA, Sporadic MVPA, and Bouted MVPA demonstrated an extremely significant correlation with osteoporosis. Increasing Total LPA, Sporadic LPA and Bouted LPA by 30 min/d, the OR values of osteoporosis decreased by 17, 13, and 19%, respectively. Similarly, augmenting Total MVPA, Sporadic MVPA, and Bouted MVPA by 10 min per day yielded more substantial reductions in osteoporosis ORs of 29, 38, and 27%, respectively. These findings align with prior research. A systematic review (36) examined the association between PA patterns and certain health outcomes, revealing that total MVPA, whether composed of Sporadic MVPA or Bouted MVPA, was positively correlated with bone health in older adults. Shephard et al. (37) assessed participants’ PA using triaxial accelerometers and the IPAQ-SF, revealing that MVPA correlates with calcaneal bone health. Engaging in 15–20 min of daily MVPA benefits bone health. MVPA is crucial for both preventing and treating osteoporosis (38). By stimulating the anabolic processes of the skeletal system, MVPA enhances bone health. Researchers examined how much of the total MVPA comprised vigorous activity and its association with all-cause mortality in middle-aged and older Australians (39). This implies that the intensity of PA within the total dose significantly influences health outcomes. Similar dose–response relationships might apply to bone health, where higher-intensity activities could notably enhance bone density and strength. Recent research indicates that engaging in MVPA for over 10 min is linked to various health indicators (7). Numerous studies have found no significant difference in health outcomes between Sporadic MVPA and Bouted MVPA (40, 41), and this study corroborates those findings.

The analysis by Chastain et al. (42), using US NHANES data, indicated that longer sedentary periods are negatively correlated with BMD in the femur and hip of older women. Gobbo et al. (43) supported older females with skeletal disorders to reduce the frequency of prolonged SED sessions exceeding 20 min and limit the duration of each SED episode to positively influence BMD. Da et al. (44) found that older adults with longer SEB duration exhibited higher osteoporosis risk, with SEB significantly increasing this risk. Conversely, osteoporosis also impacts activity levels in older adults. Not only is prolonged SED time closely associated with osteoporosis risk, but SED patterns also play a crucial role (45). Among individuals with equivalent total SED time, those who remain SED for extended periods without frequent breaks face a higher risk of osteoporosis than those who regularly stand up during SED periods (46). Therefore, the influence of SED patterns must not be overlooked when examining the relationship between SED and osteoporosis. These findings align with the study’s results. This study enhanced the SEB metric by dividing it into total SEB, 10-min, 30-min, and 60-min bouted SEB categories. With each 60 min/d increase in each indicator, all refined SEB indicators showed a significant association with osteoporosis (*p* < 0.05), with OR values rising by 34, 35, 32, and 31%, respectively. The OR value increase for osteoporosis was higher for 10 min-Bouted SEB than for total SEB, 30 min-Bouted SEB, or 60 min-Bouted SEB. The reason may be that 10 min-bouted SEB encompasses all single bouts of SED lasting over 10 min. Thus, the longer the average daily duration of 10 min-Bouted SEB, the greater its numerical impact on osteoporosis risk. Both the amount of time spent sedentary and the patterns of sedentary behavior are related to physical function (47), particularly showing significant independent associations with osteoporosis risk. Minimizing any pattern of SEB is advisable, and when necessary SEB occurs, it should not persist for extended periods to minimize health risks associated with prolonged sitting.

### 4.3 Isotemporal substitution effects of different behavioral patterns on osteoporosis in older women

The isotemporal substitution model replaces the time spent on one activity with an equivalent duration of another, while keeping the total time and other behaviors unchanged, to observe the impact of the change on the dependent variable. This model, which takes the dependencies between SEB, LPA and MVPA into account, is highly superior for exploring the reallocation of SEB, LPA and MVPA time and further developing more realistic prevention methods. In this study, replacing LPA and MVPA with 30 min of SEB significantly increased the OR values for osteoporosis in older women by 9 and 56%, respectively. These findings are consistent with previous research. Multiple studies have identified associations between the substitution of equivalent amounts of SEB for PA and various chronic diseases (48, 49). Wu et al. (50) found that replacing equivalent durations of LPA and MVPA with 30 min of SEB increased the OR values for frailty in older women by 8 and 87%, respectively. Li et al. (51) demonstrated that increasing daily SEB time by 1 h elevates frailty risk by 11.4%, and excessive SEB also negates the protective effect of PA against frailty. Replacing PA with SEB increases the risk of osteoporosis in the older adults, underscoring the need for the older adults to reduce sedentary time. Conversely, replacing SEB with PA reduces the osteoporosis risk among the older adults. Yoshioka et al. (52) examined the impact of isotemporal substitution models for PA and SEB on BMD in individuals suffering from long-term kidney disease. Results from the isotemporal substitution model indicated that substituting 10 min/d of LPA for the same duration of SDB showed no significant association with bone health, whereas substituting 10 min/d of MVPA for the same duration of SDB was favorable for strengthening bone health. According to Mitchell et al. (53), substituting 60 min per day of LPA with high-intensity PA for the same amount of time was linked to increased bone Z-scores. Conversely, substituting 60 min/d of high-intensity PA with low-intensity PA for the same duration was linked to reduced bone Z-scores, suggesting high-intensity PA benefits skeletal health. Nam et al. (54) found in their isotemporal substitution model study that replacing 30 min of SEB with an equivalent duration of LPA daily among older adults with chronic diseases reduced frailty risk. Consistent with these findings, our study replaced 30 min of SEB with LPA and MVPA, resulting in significant reductions of 8 and 47% in OR values for osteoporosis among older women, respectively. Replacing 30 min of LPA with an equivalent duration of MVPA significantly reduced the OR values for osteoporosis in older women by 28%. Zhang et al. (55) found that the relationship between daily MVPA duration and physical fitness levels in older women follows an approximate “L”-shaped curve. Specifically, as daily MVPA participation time increases, the risk of subpar physical fitness gradually decreases. However, when daily MVPA reaches approximately 30 min, the rate of risk reduction levels off. This suggests that for older women, maintaining approximately 30 min of daily MVPA may yield the most efficient physical health benefits. Although the optimal ratio between PA and SED remains undetermined, existing research indicates that both increasing PA and reducing SED time positively impact bone mineral density (56). Future studies should continue exploring the complex relationships among these factors to provide a more robust scientific foundation for health interventions.

## 5 Strengths and limitations

The advantages of this study are found in employing objective measurement methods to evaluate PA and SEB in older adults, investigating the dose–response link between PA, SEB in older women and osteoporosis, and further categorizing PA and SEB by duration. This allows for a more precise exploration of the dose–response connection between total LPA and total MVPA, as well as sporadic LPA, Bouted LPA, Sporadic MVPA, and Bouted MVPA with osteoporosis. Additionally, SDB was categorized into 10, 30, and 60 min-Bouted SEB to examine the relationship between different patterns of SEB and osteoporosis.

However, this study also has certain limitations. Although it included a substantial sample size, it unable to establish a cause-and-effect association between PA, SEB, and osteoporosis in older women. Our research team will continue to conduct long-term follow-up studies to further elucidate this causal relationship. Furthermore, all participating older women were from the same city, limiting the representativeness of their physical characteristics to the broader older women. The research team plans to conduct multicenter prospective studies to deeply explore the link between PA, SEB, and osteoporosis in older women, thereby providing more robust research evidence for developing PA and SEB guidelines for the older adults.

## 6 Conclusion

This study, based on objective behavioral monitoring and bone density assessments in older women, revealed a clear dose–response relationship and an isotemporal substitution effect between PA and SED patterns and osteoporosis. Increased sedentary time is a risk factor for the development of osteoporosis in older women. Conversely, increased total PA, Bouted PA, and Sporadic PA duration exert protective effects against osteoporosis in this population, with increased MVPA duration demonstrating the most potent protective efficacy. MVPA and LPA, when substituted for equivalent amounts of SEB (30 min/d), reduce the risk of osteoporosis in older women to varying degrees. At equivalent durations, MVPA substitution demonstrated superior protective effects against osteoporosis compared to LPA substitution. Therefore, encouraging older women to increase daily PA to replace existing SEB, minimize sedentary time, and increase MVPA may represent a more effective strategy for improving osteoporosis status.

## Data Availability

The datasets presented in this study can be found in online repositories. The names of the repository/repositories and accession number(s) can be found in the article/Supplementary material.
